# Drug Resistance Mutations Against Protease, Reverse Transcriptase and Integrase Inhibitors in People Living With HIV-1 Receiving Boosted Protease Inhibitors in South Africa

**DOI:** 10.3389/fmicb.2020.00438

**Published:** 2020-03-20

**Authors:** Adetayo Emmanuel Obasa, Sello Given Mikasi, Dominik Brado, Ruben Cloete, Kamlendra Singh, Ujjwal Neogi, Graeme Brendon Jacobs

**Affiliations:** ^1^Division of Medical Virology, Department of Pathology, Faculty of Medicine and Health Sciences, Stellenbosch University, Cape Town, South Africa; ^2^Division of Clinical Microbiology, Department of Laboratory Medicine, Karolinska Institute, Stockholm University, Stockholm, Sweden; ^3^Division of Virology, Institute for Virology and Immunobiology, Faculty of Medicine, University of Würzburg, Würzburg, Germany; ^4^South African Medical Research Council Bioinformatics Unit, South African National Bioinformatics Institute, University of the Western Cape, Bellville, South Africa; ^5^Department of Molecular Microbiology and Immunology, University of Missouri, Columbia, MO, United States; ^6^Christopher S. Bond Life Sciences Center, University of Missouri, Columbia, MO, United States

**Keywords:** HIV-1, reverse transcriptase inhibitor (RTI), protease inhibitor (PI), integrase strand-transfer inhibitor (InSTI), resistance, South Africa, resistance-associated mutations (RAMs), combination antiretroviral therapy (cART)

## Abstract

The South African national combination antiretroviral therapy (cART) roll-out program started in 2006, with over 4.4 million people accessing treatment since it was first introduced. HIV-1 drug resistance can hamper the success of cART. This study determined the patterns of HIV-1 drug-resistance associated mutations (RAMs) in People Living with HIV-1 (PLHIV-1). Receiving first (for children below 3 years of age) and second-line (for adults) cART regimens in South Africa. During 2017 and 2018, 110 patients plasma samples were selected, 96 samples including those of 17 children and infants were successfully analyzed. All patients were receiving a boosted protease inhibitor (bPI) as part of their cART regimen. The viral sequences were analyzed for RAMs through genotypic resistance testing. We performed genotypic resistance testing (GRT) for Protease inhibitors (PIs), Reverse transcriptase inhibitors (RTIs) and Integrase strand transfer inhibitors (InSTIs). Viral sequences were subtyped using REGAv3 and COMET. Based on the PR/RT sequences, HIV-1 subtypes were classified as 95 (99%) HIV-1 subtype C (HIV-1C) while one sample as 02_AG. Integrase sequencing was successful for 89 sequences, and all the sequences were classified as HIV-1C (99%, 88/89) except one sequence classified CRF02_AG, as observed in PR/RT. Of the 96 PR/RT sequences analyzed, M184V/I (52/96; 54%) had the most frequent RAM nucleoside reverse transcriptase inhibitor (NRTI). The most frequent non-nucleoside reverse transcriptase inhibitor (NNRTI) RAM was K103N/S (40/96, 42%). Protease inhibitor (PI) RAMs M46I and V82A were present in 12 (13%) of the sequences analyzed. Among the InSTI major RAM two (2.2%) sequences have Y143R and T97A mutations while one sample had T66I. The accessory RAM E157Q was identified in two (2.2%). The data indicates that the majority of the patients failed on bPIs didn’t have any mutation; therefore adherence could be major issue in these groups of individuals. We propose continued viral load monitoring for better management of infected PLHIV.

## Introduction

Exceptional improvements in combination antiretroviral therapy (cART) regimens have changed HIV/AIDS from a deadly pandemic to a chronic and manageable disease (Trickey et al., 2017). cART has made significant contributions to reducing the rates of morbidity and mortality in people living with HIV (PLHIV) and has led to better management of infection at an individual level, not only in high-income countries but also in low- and middle-income countries (Hightower et al., 2011; UNAIDS, 2017). South Africa’s national HIV cART program was introduced in 2006, with a public health approach (UNAIDS, 2018). Besides problems related to adherence, the development and spread of drug resistance have constantly challenged the long-term management of PLHIV in public health settings, where patients are often monitored using clinical or immunological parameters (Rousseau et al., 2006).

In accordance with the World Health Organization’s (WHO) guidelines, the recommended first-line cART in South Africa consists of a non-nucleoside reverse transcriptase inhibitor (NNRTI) backboned regimen of efavirenz (EFV), combined with two nucleoside reverse transcriptase inhibitors (NRTIs), namely lamivudine (3TC) and either tenofovir disoproxil fumarate (TDF), for adults, or abacavir (ABC), for children. The recommended second-line cART consists of the NRTIs zidovudine (AZT) and 3TC and a ritonavir-boosted (/r) protease inhibitor (PI), usually lopinavir (LPV/r) which was revised to atazanavir (ATV/r) in 2017 (Meintjes et al., 2017). The WHO guidelines also recommend the PI lopinavir co-formulated with ritonavir (lopinavir/ritonavir [LPV/r]) in a four-to-one ratio in first-line cART for children younger than 3 years, based on its superiority when compared with nevirapine (NVP), regardless of previous NVP exposure to prevent mother-to-child HIV transmission (Meintjes et al., 2017).

*In vitro* studies on PI-naïve PLHIV-1 infected with HIV-1 subtype C (HIV-1C) viruses, have indicated wide variations in their respective susceptibility to the PIs LPV/r and ATV/r (Sutherland et al., 2016). Observational studies from sub-Saharan Africa have shown a 14–32% prevalence of virological failure to second-line boosted PI- (bPI) based cART (Ajose et al., 2012; Sigaloff et al., 2012). In South Africa, reports of drug resistance patterns in patients receiving bPIs are scarce (Collier et al., 2017). With this study, we aimed to identify the pattern of acquired drug resistance mutations (DRMs) among PLHIV in South Africa receiving bPI second-line cART. Furthermore, we characterized the presence of primary integrase strand-transfer inhibitor (InSTI) DRMs in this specific population.

## Materials and Methods

### Ethics Statement

The study was approved by the Health Research Ethics Committee of Stellenbosch University, South Africa (N15/08/071). The study was conducted according to the ethical guidelines and principles of the Declaration of Helsinki 2013, the South African Guidelines for Good Clinical Practice and the Medical Research Council Ethical Guidelines for Research. A waiver of written informed consent was awarded to conduct sequence analyses on these samples by the Health Research Ethics Committee of Stellenbosch University, South Africa.

### Viral Load

HIV-1 Viral load was performed using the Abbott m2000sp and the Abbott m2000rt analyzers (Abbott laboratories, Abbott Park, IL, United States). RNA was isolated from patient samples according to the manufacturer’s instructions using the Abbott RealTime HIV-1 amplification reagent Kit.

### Study Design

HIV-1-positive patient samples were obtained randomly, without any knowledge of drug-resistance patterns, from the diagnostic section at the Division of Medical Virology, Stellenbosch University, and the South African National Health Laboratory Services (NHLS). Samples were collected between March 2017 and February 2018. We excluded patient samples with no previous cART regimen history and patients receiving first-line cART treatment regimen. Demographic and clinical information such as age, cART regimen, and viral load measurement (Table 1). Patients had their samples submitted for HIV-1 genotypic resistance testing to the NHLS. The NHLS provides routine genotypic antiretroviral drug resistance testing for clinics from the Western Cape, Gauteng and Eastern Cape provinces.

**TABLE 1:** Characteristics and patterns of mutations in 96 patients at the time of treatment failure.

Variable	Value
**Gender**	
Female	58 (60%)
Male	34 (35%)
Unknown	4 (4%)
Viral Load (Log_10_ copies/mL), mean (SD)	Log 8.4, 4.64 (3.02 – 6.74)
**Second-line treatment regimen**	
AZT, 3TC, LPV/r	47 (49%)
ABC, 3TC, LPV/r	17 (18%)
TDF, 3TC, LPV/r	8 (8%)
AZT, 3TC, ATV/r	6 (6%)
TDF, FTC, LPV/r	7 (7%)
Others	13 (13%)
**Major PI Mutations**	
Any PI Major Mutations	18 (19%)
>1 PI Major Mutations	17 (18%)
I47A/V	3 (3%)
I50L/V	2 (2%)
I54V	10 (10%)
I84V	7 (7%)
L76V	7 (7%)
M46I	12 (13%)
V32I	2 (2%)
V82A	12 (13%)
**Major NRTI resistance mutations**	
Any NRTI Mutations	65 (68%)
>1 NRTI Mutations	30 (31%)
M184V/I	52 (54%)
T69D	2 (2
L74V	5 (5%)
K65R/N	5 (5%)
Y115F	5 (5%)
**TAM-1 pathway**	
M41L	4 (4%)
T215Y	2 (2%)
**TAM-2 pathway**	
D67N	11 (11%)
K70R/E	17 (18%)
K219E/Q	11 (11%)
**Major NNRTI resistance mutation**	
Any NNRTI Mutations	62 (65%)
>1 NNRTI Mutations	41 (43%)
Y181C	1 (1%)
K103N/S	40 (42%)
G190A/S	10 (10%)
K101EP	6 (6%)
E138AGKQ	11 (11%)
H221Y	2 (2%)
M230L	1 (1%)
P225H	14 (15%)
V106M	13 (14%)
V108I	3 (3%)
Y188L	8 (8%)
L100I	2 (2%)
TAMS	45 (47%)
**D67N, M41L, T215Y/F, K219E/Q, K70R, and L210W**	
M184V and TAMS	15 (16%)
integrase mutations	
Major IN mutation	
T66I	1 (1%)
Y143R and T97A	2 (2%)
**IN Accessory mutations**	
E157Q	2 (2%)

*The IN mutation is based on the 89 sequences that passed the quality control.*

We included samples from children (aged below 16 years) suspected of failing on bPI – which is used as first-line therapy in children – and adults suspected of experiencing virological failure on a bPI second-line cART regimen, for which treatment information, as provided by the physicians, was available. The treatment history was collected retrospectively. The selection consisted of plasma samples (*n* = 96) obtained from patients receiving bPIs cART, according to the South African national cART guidelines (Meintjes et al., 2017). These patients are eligible for InSTI treatment consideration when PI mutations are present.

### Genotypic Resistance Testing

We performed genotypic resistance testing using viral RNA extracted from plasma. The HIV-1 protease and reverse transcriptase gene fragments were PCR-amplified using a slightly modified protocol as previously described by us (Jacobs et al., 2008). Briefly, HIV-1 protease and reverse transcriptase first-round cDNA synthesis through reverse transcription was done using amplification primers HIV-PR outer 50prot1 (5′-TAA TTT TTT AGG GAA GAT CTG GCC TTC C-3′) and HIVRT outer Mj4 (5′-CTG TTA GTG CTT TGG TTC CTC T–3′), position 2085-2109 and 3399-3420 of the HXB2 reference numbering, with an expected fragment size of approximately 1314 base pairs (bps) (Plantier et al., 2006). For second-round PCR amplification, primers 50prot2 (5′-TCA GAG CAG ACC AGA GCC AAC AGC CCC A–3′) and NE13 (5′-CCT ACT AAC TTC TGT ATG TCA TTG ACA GTC CAG CT–3′), position 2136–2163 and 3334–3300 of the HXB2 reference numbering, with an expected fragment size of approximately 1300 bps, were used (Plantier et al., 2006). The integrase gene fragment amplification steps were performed as previously described by us (Brado et al., 2018). Sequencing reactions were performed as previously described by us (Brado et al., 2018). As part of quality control, each of the viral sequences was inferred on a phylogenetic tree in order to eliminate possible contamination. DRMs were interpreted using the Stanford University HIV Drug Resistance Database version 8.7^1^. Subtyping was carried out using REGAv3 and COMET (Pineda-Peña et al., 2013). Phylogenetic analysis was carried out using MEGAv6.

## Results

We included patients receiving bPI as part of their cART regimens. We confirmed the successful amplification and Sanger sequencing of the protease, reverse transcriptase and integrase gene fragments of the HIV-1 polymerase gene for 96 samples. In the Integrase region seven sequences did not pass the quality control. Those sequences were excluded from the final analyses. Among the patients, 4% (4/96) of the samples did not indicate their patient file as being either male or female. Hence, they were classified as unknown. The ages ranged from 2 to 66 years.

Seventeen (*n* = 17; 18%) of the patients were 16 years or younger. Of these patients, three (*n* = 3; 3%) were female, 12 (13%) were male, while for two (2%) the gender had not been disclosed by the physician. The NRTI cART regimen combinations administered were ABC plus 3TC (*n* = 6; 6% of patients), AZT plus 3TC (*n* = 6; 6%), stavudine [d4T] plus 3TC (*n* = 1; 1%), TDF plus emtricitabine [FTC] (*n* = 2; 2%), and TDF plus AZT (*n* = 1; 1%). Fourteen (15%) had received LPV/r, two (2%) had received ATV/r and one (1%) had received darunavir (DRV/r) as their bPIs.

We had a total of 76 adults; 55 (57%) were women, and 22 (23%) were men, while with two (2%) the gender was not disclosed by the physician. The most commonly used NRTI combination was AZT plus 3TC (*n* = 56; 58%), compared with those receiving ABC plus 3TC (*n* = 11; 11%) and TDF plus 3TC (*n* = 8; 8%). Other regimens given were d4T plus 3TC (*n* = 2; 2%), TDF plus FTC (*n* = 2; 2%), TDF plus etravirine [ETR] (*n* = 1; 1%) and AZT plus TDF (*n* = 1; 1%). Fifty-eight (60%) had received LPV/r and six (6%) had received ATV/r as their bPIs and three (3%) are currently receiving DRV/r.

### HIV-1 Subtyping

**FIGURE 1:**
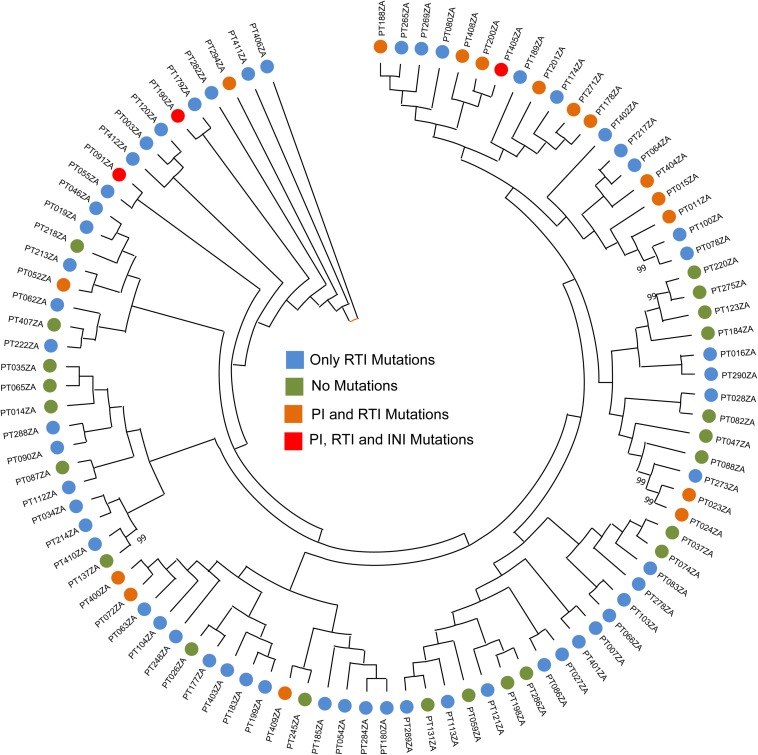
Neighbor-joining phylogenetic tree. The phylogenetic analysis is carried out using the PR/RT sequences. Bootstrap support > 70% was shown.

Subtyping was carried out using REGAv3 and COMET. While REGAv3 provide subtyping for all the RT/PR sequences as well as IN sequences, COMET failed to subtype the IN sequences as the majority of the IN sequences were typed as CPZ. Therefore we used HIV-1 BLAST to identify the nearest subtype for the IN sequences. Based on the PR/RT sequences (*n* = 96), 99% were identified as HIV-1C while one as 02_AG (PT405ZA). Based on the IN sequences (*n* = 89), patient samples PT405ZA identified as 02_AG while 88 (99%) samples as HIV-1C. The subtyping data is presented in Supplementary Table S1. We also performed phylogenetic analysis to identify any specific clusters. The neighbor-joining phylogenetic tree did not identify any specific cluster of a transmission network (Figure 1).

### NRTI Resistance-Associated Mutations

Table 1 shows the number of resistance-associated mutations (RAMs) observed among the 96 sequences analyzed. We observed M184V/I as the most prevalent NRTI mutation. M184V/I was detected in 52 (54%) patients suspected of failing cART. Of these, 36 (69%) patients were receiving a combination of AZT plus 3TC, compared with 13 (14%) patients receiving an ABC plus 3TC combination. M184V/I was also found in two (4%) patients receiving TDF plus 3TC, compared with those receiving FTC plus TDF (*n* = 1; 2%). L74V was detected in five (5%) patients – three (3%) patients receiving ABC plus 3TC, and two (2%) patients receiving AZT plus 3TC. The K65R/N mutation occurred in five (5%) patients; K65R occurred in two (2%) patients receiving AZT plus 3TC, and in one (1%) patient receiving TDF plus FTC. The Y115F mutation occurred in five (5%) patients. However, Y115F occurred more often in patients receiving AZT plus 3TC (*n* = 4; 4%); it occured in one (1%) patient receiving ABC plus 3TC. The A62V mutation occurred in one (1%) patient receiving AZT plus 3TC. V75I occurred in one (1%) patient receiving AZT plus 3TC.

Thymidine analog mutations (TAMs) were grouped into TAMs1 and TAMs2. The most frequent TAMs observed were K70R/E in 10 (10%) patients receiving AZT plus 3TC, in three (3%) patients receiving ABC plus 3TC, and in one (1%) patient receiving FTC plus TDF. D67N occurred in five (5%) patients receiving AZT plus 3TC and in three (3%) patients receiving ABC plus 3TC. D67N occurred in one (1%) patient receiving both TDF plus 3TC and an FTC plus TDF-based regimen. The K219E/Q mutation occurred in six (6%) patients receiving AZT plus 3TC, and in two (2%) patients receiving FTC plus TDF. The M41L mutation occurred in three (3%) patients receiving AZT plus 3TC, and in one (1%) patient receiving 3TC plus ATV/r. T215Y occurred in two (2%) patients receiving AZT plus 3TC.

### NNRTI Resistance-Associated Mutations

Table 1 shows the number of NNRTI RAMs observed. We observed that the K103N/S mutation occurred in 41 (43%) of those patients failing cART. Of the patients with this mutation, 24 (59%) patients were receiving AZT plus 3TC, and five (5%) patients were receiving ABC plus 3TC. P225H occurred in 10 (10%) patients receiving AZT plus 3TC, in three (3%) patients receiving ABC plus 3TC, and in two (2%) patients receiving TDF plus 3TC. V106M occurred in 10 (10%) patients receiving AZT plus 3TC, and in two (2%) patients receiving ABC plus 3TC. The patients might have received the EFV or NVP based regiments as their first-line treatment though the past treatment history was not available.

Y188L occurred in five (5%) patients receiving AZT plus 3TC, and in two (2%) patients receiving TDF plus ATV/r, and in one (1%) patient receiving ABC plus 3TC. G190G/A occurred in six (6%) patients receiving AZT plus 3TC, and in three (3%) patients receiving ABC plus 3TC. K101EP occurred in three (3%) patients receiving AZT plus 3TC, and in one (1%) patient receiving ABC plus 3TC.

E138QGA occurred in four (4%) patients receiving AZT plus 3TC, in three (3%) patients receiving ABC plus 3TC, and in one (1%) patient receiving TDF plus 3TC. V108I occurred in one (1%) patient receiving AZT plus 3TC. H221Y and M230L occurred in one (1%) patient each; both these patients were receiving AZT plus 3TC.

### PI Resistance-Associated Mutations

Of the 96 patients, 75 (81%) were receiving the LPV/r-containing regimen, followed by the ATV/r- (*n* = 8; 8%) and DRV/r- (*n* = 4; 4%) containing regimens. We identified 18 (18%) patients with major PI RAMs. Of those, a substantial majority of 16 (89%) patients were receiving LPV/r as their bPI regimen, while two (11%) patients were receiving ATV/r (Table 1). The most common major PI RAMs observed were M46I and V82A (*n* = 12; 12%); I54V (*n* = 10; 10%); I84V and L76V (*n* = 7; 7%); I47A/V (*n* = 3; 3%); I50L/V (*n* = 2; 2%); and V32I (*n* = 2; 2%) (Table 1).

### InSTIs Resistance-Associated Mutations

**FIGURE 2:**
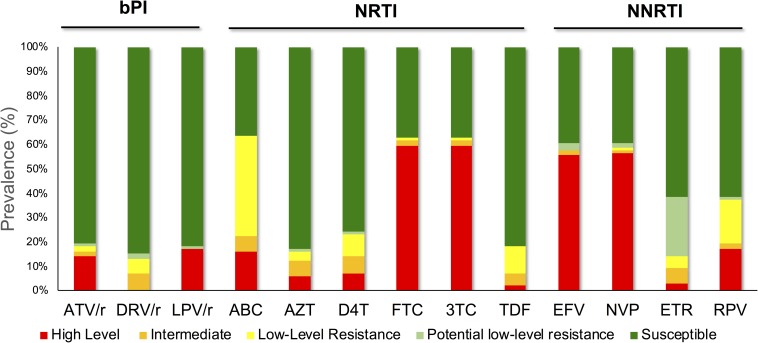
The observed resistance in patients receiving bPIs as part of their cART. High-level resistance was shown in 17 (17%) and 14 (14%) of PLHIV against LPV/r and (ATV/r), respectively, while seven (7%) showed intermediate cross-resistance to DRV/r. Despite off-treatment with NNRTIs, more than half of the patients were shown to have high-level resistance to NVP (57%, *n* = 56) and EFV (56%, *n* = 55).

We successfully sequence 89 INI samples. In our cohort, we identified major InSTI RAMs in patients who had received the DRV/r-containing regimen. Two (2%) patient had a viral sequences with Y143R major InSTI mutation in combination with the accessory T97A mutation, which confers high-level resistance to raltegravir (RAL), intermediate resistance to elvitegravir (EVG), and potential low-level resistance to bictegravir and dolutegravir (DTG). A patient receiving AZT, 3TC, EFV, d4T and LPV/r had a virus sequence with the T66I mutation (Table 1). The mutation identified and classified as an ‘accessory’ integrase, E157Q, occurred in two (2%) patients. One patient was receiving EFV, TDF and 3TC and the other AZT, 3TC and ATV/r. The level of drug resistance against all the cARTs is presented in Figure 2. The observed RAMs in patient receiving bPI based treatment regimen shows high-level resistance was demonstrated in 17 (17%) and 14 (14%) of PLHIV against LPV/r and (ATV/r), respectively, while seven (7%) showed intermediate cross-resistance to DRV/r. Despite off-treatment with NNRTIs, more than half of the patients were shown to have high-level resistance to NVP (57%, *n* = 56) and EFV (56%, *n* = 55). High-level resistance were also observed with patients receiving NRTI-based treatment to FTC (60%, *n* = 59) and 3TC (60%, *n* = 59).

## Discussion

In this study, we analyzed 96 HIV-1 RT/PR and 89 IN sequences from PI-experienced and InSTI-naïve patients for major RAMs. The patients were being treated with a bPI second-line cART regimen and were suspected of virological failure. Second-line cART consisted of two NRTIs, backboned by a PI, if previously treated with an NNRTI-based regimen, and vice versa.

As expected, major DRMs against NRTIs and NNRTIs were present at a rate of 65% (*n* = 65) and 62% (*n* = 62), respectively. Despite being on a bPI, only 18% (*n* = 18) of our study sequences harbored major PI RAMs. This is in line with a previous study conducted in Sweden, where it was predicted that HIV-1 subtype C would be more prone to failure in bPIs (Amanda et al., 2016). We identified 27 (27%) sequences not showing any DRM against the drug classes mentioned above and therefore could indicate a problem of poor adherence, rather than the selection of resistant variants.

M184V, the most common NRTI RAM, occurred more frequently in patients receiving AZT plus 3TC, in comparison with patients receiving the ABC plus 3TC regimen. Our findings correspond with previous studies conducted in South Africa with PLHIV, showing M184V/I as the most prevalent NRTI mutation (Marconi et al., 2008; Van Zyl et al., 2011, 2013; Wallis et al., 2011; Lombaard et al., 2016; Neogi et al., 2016; Steegen et al., 2016a, b; Rossouw et al., 2017; Penrose et al., 2018). The K65R and Y115F RAMs occurred more frequently in patients receiving AZT plus 3TC, rather than in patients receiving ABC plus 3TC. TAMS 1 and 2 pathway mutations occurred more frequently in patients receiving the AZT plus 3TC cART regimen but were low in patients receiving ABC plus 3TC, TDF plus 3TC, and FTC plus TDF. The most frequent TAM was K70R/E, which occurred mostly in patients receiving AZT plus 3TC, as opposed to ABC plus 3TC and FTC plus TDF. In our study, M184V, L74V, K65R, and Y115F were the most common major NRTI RAMs in patients receiving LPV/r as their bPIs.

The K103N mutation occurred at a higher frequency in patients receiving AZT plus 3TC or ABC plus 3TC than in those receiving TDF plus 3TC, and 3TC plus d4T. The high rate of K103N RAM is also well documented and has been observed in several previous studies^–^(Bronze et al., 2012; Steegen et al., 2017). The group receiving AZT plus 3TC or ABC plus 3TC showed the highest rates of NNRTI mutations such as P225H, V106M, E138A/G/K/Q, G190A/S, and Y188L occurred most frequently in patients receiving AZT plus 3TC or ABC plus 3TC. The presence of NNRTI RAMs when they were off the NNRTI indicating either the carryover NNRTI RAMs from the failed first-line therapy or could be the transmitted DRMs in children from the mother due to vertical transmission prophylaxis.

The majority of our patients were receiving LPV/r as their bPIs. The M46I and V82A RAMs were the most common mutations observed in patients receiving LPV/r compared with ATV/r. We identified a low frequency of M46I and V82A in patients receiving ATV/r as their bPIs. The group receiving AZT plus 3TC or ABC plus 3TC showed the highest rate of PIs such as I54V, I84V, L76V, I47A/V, I50L/V, and V32I. Our findings are in agreement with Neogi et al.’s (2016) study where major PI RAMs were observed in 5% of patients; among them, V82A 65% (28/43), I54V 63% (27/43), L76V 23% (10/43), and L90M 16% (7/43) were the most frequent (Neogi et al., 2016). Our findings also showed M46I and V82A RAMs as the most prevalent major PI RAMs. Furthermore, these findings are in agreement with Rossouw et al. (2015) that observed both V82A and M46I has the most common mutation in infected children receiving PI-based cART. Chimbetete et al. (2018) observed similar results, with all three drug classes showing their DRMs at similar rates. The most common PI RAM reported by Chimbetete et al. (2018) was M46I 28 (33%), followed by I50V 18 (21%) and V82A 18 (21%). We observed more high-level resistance to patients receiving LPV/r compared with ATV/r and DRV/r. Van Zyl et al. (2013) findings are consistent with ours, as they also identified more high-level resistance in patients receiving LPV/r compared with those receiving ATV/r and DRV/r.

We analyzed the integrase gene for the presence of treatment-compromising polymorphisms and DRMs against InSTIs. We observed the presence of Y143R in combination with T97A in one of our patients receiving ABC, 3TC, LPV/r. N155H, Q148H/R/K, and Y143R/C/H are the three major recognized pathways of genotypic resistance against InSTIs (Doyle et al., 2015). We confirmed Y143R in our study and this mutation in combination with T97A also impaired EVG susceptibility and showed possible low-level resistance. Furthermore, our data suggest that EVG activity is compromised in the presence of any RAL RAM, in this case Y143R. We also identified the presence of E157Q in 2 (2%) patients. The presence of this mutation is concerning, as these mutations are associated with potential low-level resistance to both RAL and EVG. In a previous study conducted by Brado et al. (2018), we also found E157Q on HIV-1-infected South African sequences retrieved from the HIV database. Viruses having E157Q were found to be associated with treatment failure of a DTG-containing regimen (Brado et al., 2018). A study has shown that eight patients who had the E157Q mutation and were initiated with DTG-based therapy did not experience viremia suppression below detection level (Neogi et al., 2018).

Furthermore, we identified the presence of T66I mutation in 1 (1%) patient. T66I confers low-level resistance to RAL and high-level resistance to EVG. The low prevalence of DRMs to InSTIs in our cohort should not be underestimated. RAMs against InSTI raises the question about the positioning of DTG in the treatment guidelines for South Africa. Previous studies have shown that a considerable minority of patients develop cross-resistance to DTG after exposure to RAL and EVG; resistance to DTG has not yet been reported in patients from South Africa (Fourati et al., 2015; Mesplède and Wainberg, 2015). As DTG was proposed as the first-line drug, it is essential to conduct studies in real-life clinical settings to identify the efficacy of DTG as limited viral load monitoring and without drug resistance genotyping may compromise the next-generation InSTIs to be used.

Our study had some limitations that merit comments. First, the sample size was small compared to the total number of patients who are receiving cART in South Africa. However, to the best of our knowledge, there has not been any statistical study that reports on the number of patients receiving second-line cART from South Africa. Second, the majority of our patients were receiving LPV/r as their bPIs compared to other bPI regimens. We cannot tell for certain whether the patients having RAL resistance according to the sequence have had access to an RAL-based treatment regimen. Finally, we did not have any adherence data for these patients and the DRM data were only based on population sequencing, therefore we could not detect minor mutations below 20% of the population.

## Conclusion

We identified patterns of RAMs against reverse transcriptase inhibitors and PIs from patients suspected of failing on the South African second-line national cART program. Very low or no primary InSTI RAMs were detected in second-line failure patients. The majority of them had M184V mutations that could have been carried over from the first-line cART. Given the negative effect of M184V mutations on viral fitness, it is more plausible to the recycling of 3TC/FTC in second-line cART maintains the presence of M184V. The non-identification of any RAMs in one-third of the patients and the presence of PI RAM in only one-fifth of the patients indicate that the failure may not be due to RAM, but might be due to adherence. Given the limited cART drug availability and high public health burden, we propose for genotypic resistance testing should be performed before switching to InSTIs-based regimen in our setting. This will not only detect treatment failure earlier but will also identify poor treatment adherence. Data generated from this study can assist in the development of cART guidelines for patients who experience treatment failure in resource-limited settings where genotyping is not available. Studies that address operational issues, such as the optimal use of treatment monitoring tools, should be a research priority.

## Data Availability Statement

The raw data supporting the conclusions of this article will be made available by the authors, without undue reservation, to any qualified researcher.

## Ethics Statement

The study was approved by the Health Research Ethics Committee of Stellenbosch University, South Africa (N15/08/071). The study was conducted according to the ethical guidelines and principles of the Declaration of Helsinki 2013, the South African Guidelines for Good Clinical Practice and the Medical Research Council Ethical Guidelines for Research. A waiver of written informed consent was awarded to conduct sequence analyses on these samples by the Health Research Ethics Committee of Stellenbosch University, South Africa.

## Author Contributions

GJ and UN conceptualized and designed the study. AO performed the laboratory experiments, detailed the sequence analyses, and wrote the first draft of the manuscript. SM helped with demographic data and sample collection. UN performed the sequencing experiments and the sequence analyses. DB, RC, and KS helped with manuscript proofreading and editing. All authors read and approved the final manuscript.

## Conflict of Interest

The authors declare that the research was conducted in the absence of any commercial or financial relationships that could be construed as a potential conflict of interest.

## Footnotes

1 https://hivdb.stanford.edu/

## Supplementary Material

TABLE S1 The subtyping data is presented in Supplementary Table S1. Subtyping was carried out using REGAv3 and COMET. Based on the PR/RT sequences (*n* = 96), 99% were identified as HIV-1C while one as 02_AG (PT405ZA). Based on the IN sequences (*n* = 89), patient samples PT405ZA identified as 02_AG while 88 (99%) samples as HIV-1C.
